# Dual DNA Transfection Using 1,6-Hexanedithiol-Conjugated Maleimide-Functionalized PU-PEI_600_ For Gene Correction in a Patient iPSC-Derived Fabry Cardiomyopathy Model

**DOI:** 10.3389/fcell.2021.634190

**Published:** 2021-08-04

**Authors:** Chian-Shiu Chien, Yueh Chien, Yi-Ying Lin, Ping-Hsing Tsai, Shih-Jie Chou, Aliaksandr A. Yarmishyn, Elham Rastegari, Ting-Xian Wang, Hsin-Bang Leu, Yi-Ping Yang, Mong-Lien Wang, Ying-Chun Jheng, Henkie Isahwan Ahmad Mulyadi Lai, Lo-Jei Ching, Teh-Ia Huo, Jong-Yuh Cherng, Chien-Ying Wang

**Affiliations:** ^1^Department of Medical Research, Taipei Veterans General Hospital, Taipei, Taiwan; ^2^School of Medicine, National Yang-Ming Medical University, Taipei, Taiwan; ^3^Institute of Pharmacology, National Yang-Ming University, Taipei, Taiwan; ^4^Department of Chemistry and Biochemistry, National Chung-Cheng University, Chiayi County, Taiwan; ^5^Heath Care and Management Center, Taipei Veterans General Hospital, Taipei, Taiwan; ^6^School of Pharmaceutical Sciences, Institute of Food Safety and Health Risk Assessment, National Yang-Ming University, Taipei, Taiwan; ^7^Department of Medical Laboratory, Faculty of Health Sciences, University of Selangor, Selangor, Malaysia; ^8^Division of Gastroenterology, Department of Internal Medicine, Taipei Veterans General Hospital, Taipei, Taiwan; ^9^Division of Trauma, Department of Emergency Medicine, Taipei Veterans General Hospital, Taipei, Taiwan

**Keywords:** Fabry disease, cardiomyopathy, induced pluripotent stem cells, polyurethane, polyethyleneimine, CRISPR/Cas9, GLA

## Abstract

Non-viral gene delivery holds promises for treating inherited diseases. However, the limited cloning capacity of plasmids may hinder the co-delivery of distinct genes to the transfected cells. Previously, the conjugation of maleimide-functionalized polyurethane grafted with small molecular weight polyethylenimine (PU-PEI_600_-Mal) using 1,6-hexanedithiol (HDT) could promote the co-delivery and extensive co-expression of two different plasmids in target cells. Herein, we designed HDT-conjugated PU-PEI_600_-Mal for the simultaneous delivery of CRISPR/Cas9 components to achieve efficient gene correction in the induced pluripotent stem cell (iPSC)-derived model of Fabry cardiomyopathy (FC) harboring *GLA* IVS4 + 919 G > A mutation. This FC *in vitro* model recapitulated several clinical FC features, including cardiomyocyte hypertrophy and lysosomal globotriaosylceramide (Gb3) deposition. As evidenced by the expression of two reporter genes, GFP and mCherry, the addition of HDT conjugated two distinct PU-PEI_600_-Mal/DNA complexes and promoted the co-delivery of sgRNA/Cas9 and homology-directed repair DNA template into target cells to achieve an effective gene correction of IVS4 + 919 G > A mutation. PU-PEI_600_-Mal/DNA with or without HDT-mediated conjugation consistently showed neither the cytotoxicity nor an adverse effect on cardiac induction of transfected FC-iPSCs. After the gene correction and cardiac induction, disease features, including cardiomyocyte hypertrophy, the mis-regulated gene expressions, and Gb3 deposition, were remarkably rescued in the FC-iPSC-differentiated cardiomyocytes. Collectively, HDT-conjugated PU-PEI_600_-Mal-mediated dual DNA transfection system can be an ideal approach to improve the concurrent transfection of non-viral-based gene editing system in inherited diseases with specific mutations.

## Introduction

The clustered regularly interspaced short palindromic repeats/CRISPR-associated protein-9 nuclease (CRISPR/Cas9) is a powerful gene editing system that can generate point mutations, deletions and insertions in mammalian cells. CRISPR/Cas9 system has been widely applied for genetic manipulation and biomedical research, for example, it was used to disrupt the *Pcsk9* gene *in vivo* to reduce blood cholesterol level and protect against cardiovascular disease (Ding et al., 2014). In our previous research, we used CRISPR/Cas9 editing to perform gene knockout to model inherited diseases *in vitro* (Song et al., 2016), or to correct the mutated genes and aberrant phenotypes in disease models (Huang et al., 2019; Yang et al., 2020a). The effectiveness of CRISPR/Cas9-mediated gene editing generally depends on the precise delivery and co-expression of single guide RNA (sgRNA), Cas9, and homology-directed repair (HDR) DNA template to the same target cell. However, due to the limited cloning capacity of plasmids, DNA template and Cas9-encoding gene with relatively large size, are usually transfected separately into target cells, thus decreasing the probability of their co-expression and successful gene editing. Hence, a more efficient method for the co-delivery of multiple genes into target cells is in demand to improve the current transfection methodologies.

Non-viral gene delivery with low cytotoxicity and immunogenicity has been widely applied as a safer delivery system than viral-mediated delivery (Boulaiz et al., 2005). The design of nanoparticle vehicles that are highly biocompatible and can efficiently deliver DNA is usually crucial for gene therapy methods to treat human diseases. Recently, we designed cationic polyurethane nanoparticles grafted with small molecular weight (Mw = 600) polyethylenimine (PU-PEI_600_) (Hung and Cherng, 2015) with high transfection efficiency and low cytotoxicity. Furthermore, we functionalized PU-PEI_600_ with maleimide (PU-PEI_600_-Mal), which allows covalent conjugation of separately prepared complexes with different DNA species (Hung and Cherng, 2015). Such conjugation significantly increased co-expression of genes encoded in these two plasmids in a single cell. Therefore, in this study, we sought to apply this system for the co-delivery of CRISPR/Cas9 components encoded in two different plasmids.

Fabry disease is an inherited lysosomal storage disorder caused by the deficiency of α-galactosidase A (GLA), the enzyme responsible for the cleavage of globotriaosylceramide (Gb3) (Eng et al., 1994). The cardiac manifestation of Fabry disease, also known as Fabry cardiomyopathy (FC), is characterized by ventricular hypertrophy and conduction abnormalities that may progress to congestive heart failure or other life-threatening cardiac events (Karras et al., 2008). In Taiwan, the majority of FC patients carry the unique GLA IVS4 + 919 G > A mutation and exhibit late-onset cardiac manifestation (Hsu et al., 2014; Lee et al., 2014). For the treatment of FC, the enzyme replacement therapy (ERT) is considered to be the standard therapy. However, although early administration of ERT has shown efficacy in correcting symptoms of FC, including left ventricular hypertrophy (Weidemann et al., 2009) and cardiac dysfunction (Yousef et al., 2013), the uncontrolled progress of the disease is still a major concern (Yousef et al., 2013). Therefore, an advanced therapeutic strategy is required to ameliorate the disease progression or to improve the treatment outcome in FC.

Previously, we have generated FC patient-specific induced pluripotent stem cells (iPSCs) and differentiated them into iPSC-derived cardiomyocytes (iPSC-CMs) (Chou et al., 2017). Remarkably, these iPSC-CMs that carried GLA IVS4 + 919 G > A mutation, recapitulated FC-specific features such as Gb3 deposition and cardiomyocyte hypertrophy (Chien et al., 2016, 2018). Thus, these patient-derived cells can be applied as an *in vitro* disease model for evaluating the efficacy of gene editing therapy. In the present study, we generated patient-specific iPSC-CMs from a carrier of GLA IVS4 + 919 G > A mutation who was diagnosed with FC. These iPSC-CMs were characterized by disease-specific phenotypes and used as the disease model (Chien et al., 2016, 2018). We demonstrated that the HDT-conjugated PU-PEI_600_-Mal could successfully increase the co-delivery of DNA template approach and CRISPR/Cas9 gene editing machinery leading to ameliorating FC-associated phenotypes.

## Materials and Methods

### Synthesis of Polyurethane (PU)

PU was synthesized by mixing 0.3 mL L-lysine-diisocyanate (LDI; Alfa Aesar, Haverhill, MA) and 130 mg N,N′-bis-(2-hydroxyethyl)-piperazine (PPA; Alfa Aesar) in 1 mL anhydrous dimethylformamide (DMF) inside a three-neck reaction flask under a dry nitrogen purge. The reaction was performed at 70°C for 12 h using 0.5% dibutyltin dilaurate as a catalyst. Then, ethanol was slowly added into the reaction mixture until no unreacted LDI was detected. The PU was collected after precipitation/purification in ethyl ether and vacuum-drying at 40°C. The characteristics of PU were confirmed by ^1^H NMR (400 MHz, DMSO-d6, ppm).

### Synthesis of PU-PEI_600_ and PU-PEI_600_-Mal

For the synthesis of PU-PEI_600_, 100 mg PU and an excess amount (300 mg) of PEI_600_ (Sigma-Aldrich, St. Louis, MO) were dissolved in dichloromethane (DCM) in a round-bottom flask and were left to react at 75°C. After stirring for 48 h, the free PEI_600_ in DCM was removed and the precipitate (PU-PEI_600_) was collected. PU-PEI_600_ was further washed three times with DCM to remove the unreacted PEI_600_. Distinct signals of ^1^H NMR (400 MHz, D2O, ppm) from the grafting of PEI_600_ appeared at δ: 0.95 (–NHCH2CH2NH–), 2.0 (–CONHCH2CH2–), 2.38, 3.12 (–NHCH2CH2NH–), and 3.8 (–CONHCH2CH2–).

For functionalization with maleimide, 5 mg of 3-maleimidopropionic acid (TCI, Tokyo, Japan) dissolved in DCM was mixed with 4 mg N-(3-dimethylaminopropyl)-N’-ethylcarbodiimide hydrochloride (EDC; Alfa Aesar) and 4 mg N-hydroxysuccinimide (NHS; Alfa Aesar) and allowed to react for 30 min at 35°C. Then, this mixed solution (containing 3-(maleimido)propionic acid N-hydroxysuccinimide ester) was reacted with 200 mg PU-PEI_600_ dissolved in 1 mL of methanol to form PU-PEI_600_-Mal in 12 h at room temperature. The product was vacuum-dried and rinsed with acetonitrile (ACN). PU-PEI_600_-Mal was also analyzed by ^1^H NMR. The new signals resultant from grafting of maleimide in ^1^H NMR (400 MHz, D2O, ppm) were observed at δ: 2.94, 4.02 (–COCH2CH2N(CO–)2), 5.05 (–CH2CH2NHCOCH2CH2–), and 8.22 (–COCH = CHCO–).

### Preparation of PU- PEI_600_-Mal/DNA Complexes

PU-PEI_600_-Mal was dissolved in 20 mM HEPES buffer (pH 7.4) to a concentration of 10 mg/mL. PU-PEI_600_-Mal/plasmid DNA complexes were individually prepared at PU-PEI_600_-Mal/DNA ratio of 10/1 (w/w) with the final concentration of DNA in the complexes of 5 μg/mL. These polymer/DNA complexes were self-assembled in a HEPES buffer at room temperature for at least 30 min.

### Preparation of Maleimide-Thiol Conjugates of PU-PEI_600_-Mal/DNA Complexes

For dual DNA transfection, two distinct PU-PEI_600_-maleimide/DNA complexes were separately prepared and conjugated in the presence of 1,6-hexanedithiol (HDT) dissolved in HEPES buffer. The conjugation reaction was conducted with HDT/PU-PEI_600_-Mal molar ration of 1.25/1 for 30 min at room temperature.

### Measurement of the Sizes of Polymer/DNA Complexes by Dynamic Light Scattering

The hydrodynamic sizes of polymer/DNA complexes and their conjugates were measured by dynamic light scattering (DLS) using a Nicomp 380 Submicron Particle Sizer (PSS, Santa Barbara, CA, United States) at 25°C with a 5 mW, λ = 633 nm He–Ne laser as an incident beam.

### CRISPR/Cas9 Design and Preparation of Plasmid DNA

sgRNA-coding sequence (ACAAATACTTCCAAATAGTGTGG) and HDR DNA template sequence (chrX:101,399,138-101,400,132; GRCh38/hg38 assembly) were cloned into pLAS2w.Ppuro (Academia Sinica, Taipei, Taiwan) encoding RFP reporter. pSpCas9(BB)-2A-GFP (PX458) (Addgene, Watertown, MA, United States) encoding GFP reporter was used for Cas9 expression. The plasmids were amplified in Escherichia coli (DH5α strain) and purified by column chromatography using a Plasmid Mega Kit (QIAGEN, Hilden, Germany). The purity of each DNA sample was determined by the ratio of UV absorbance at 260/280 nm and DNA was stored in Tris-EDTA buffer (10 mM Tris and 1 mM EDTA, pH 8.0).

### iPSC Generation

The peripheral blood mononuclear cells (PBMCs) were taken from Fabry patients and further isolated by gradient centrifugation in Histopaque-1077 (Sigma Aldrich). PBMCs were collected from the buffy coat and incubated in X-VIVO medium (Lonza, Basel, Switzerland). PBMCs were transfected with reprogramming factors (OCT4, SOX2, KLF4, L-MYC, LIN28, and TP53shRNA) and enhancer factor (EBNA1) using the Nucleofector Kits for Human T Cells (Lonza). After transfection, the cells were plated onto inactivated MEF-coated dishes with hESC medium (DMEM/F12 with 20% KSR, 1% GlutaMAX, 1% NEAA, 1% Penicillin/Streptomycin, 0.1 μM 2-mercaptoethanol, and 10 ng/mL bFGF) supplemented with 0.5 μM Thiazovivin, 2 μM SB431542, 0.5 μM PD0325901, and 50 μM Vitamin C. The cells were incubated at 37°C and supplied with 5% CO_2_ for ∼14–30 days until colony formation and medium was changed every 2 days.

### Differentiation of iPSCs to Cardiomyocytes

FC-iPSCs and control-iPSCs were differentiated into cardiomyocytes (CMs) according to the reported established protocol (Lian et al., 2013; Chou et al., 2017). FC-iPSCs or control-iPSCs were first seeded on Geltrex-coated plates in mTeSR1 medium (STEMCELL Technologies, Vancouver, Canada), and Accutase (Innovative Cell Technologies, San Diego, CA, United States) was used to detach iPSCs from the plates. The iPSCs were then re-suspended in mTeSR1 with 5 μM of Y27632 (Tocris Bioscience, Bristol, United Kingdom), a ROCK inhibitor, and re-plated on Geltrex-coated 12-well plates at a density of 1 × 10 (Hung and Cherng, 2015) cells per well. After 4 days of culturing in mTeSR1 medium, the culture medium was changed to RPMI (Thermo Fisher Scientific) supplemented with B-27 supplement minus insulin (Thermo Fisher Scientific) and CHIR99021 (Selleck Chemicals, Houston, TX), a GASK3 inhibitor. After 24 h, the medium was replaced with RPMI/B-27 minus insulin. On day 3 of differentiation, combined medium was prepared by mixing the old medium with fresh RPMI/B-27 minus insulin at a 1:1 ratio. The medium was replaced with combined medium containing 5 μM of IWP-2 (Tocris Bioscience, Bristol, United Kingdom), a Wnt signaling inhibitor. On day 5 of differentiation, the medium was changed to fresh RPMI/B-27 minus insulin. On day 7 of differentiation, RPMI with B-27 was added and changed every other days for the following 3 weeks. CMs appeared after 3 weeks, showing regular contractile movements.

### Immunofluorescent Staining

Cells were fixed with 1% paraformaldehyde solution at room temperature for 15 min and permeabilized with 0.1% Triton X-100 for 10 min. After several washes with 1 × PBS, fixed cells were blocked with 3% BSA and 5% FBS at room temperature for 1 h and incubated with primary antibody against Gb3/CD77 (1:100, ab19795, Abcam, Cambridge, United Kingdom) or cTnT (xxx) overnight at 4°C. Cells were washed three times with 1 × PBS and incubated with secondary antibodies at room temperature for 1 h. Finally, the cells were mounted and observed using a fluorescent or FV10i confocal microscope (Olympus, Center Valley, PA, United States). The measurement of cardiomyocyte size was conducted by the measurement of the cellular area contents of iPSC-derived cardiomyocytes. Cardiomyocytes were then plated onto gelatin-coated dishes and subjected to subsequent analysis. The cellular images of cTnT-positive cells were recorded using the confocal microscope (FV10i, Olympus) at various time points (i.e., 30, 40 and 60 days after cardiomyocyte differentiation). The cellular area pixels of cTnT-positive cells was analyzed with the ImageJ software package (National Institutes of Health, Bethesda, MD, United States).

### Transmission Electron Microscopy (TEM)

The morphological characterization of iPSC-derived cardiomyocytes was performed by using a JEM-2000 EX II transmission electron microscope (JEOL, Tokyo, Japan). The cardiomyocytes were dehydrated and embedded in epoxy resin for ultra-thin sectioning. The ultra-thin sections were covered with 400 mesh carbon-coated copper TEM grid. The grid was stained with 1% phosphotungstic acid (Sigma-Aldrich, St. Louis, MO, United States) for 20 min and tapped with filter paper to remove the excess water. The samples were air-dried overnight and then observed under TEM.

### Cell Viability Assay

The cytotoxicity of PU-PEI_600_-Mal/DNA complexes was assessed utilizing the MTT assay. iPSCs were seeded in complete DMEM with a seeding density of 1 × 10^4^ cells/well in a 96-well plate. The cells were incubated in 5% CO2 at 37°C for 24 h. Afterward, the PU-PEI_600_-Mal/DNA complexes were prepared in FBS-free DMEM medium and added to iPSCs at a volume of 200 μL/well. After incubating for 1 h, the iPSCs were washed with PBS and supplemented with complete DMEM medium containing FBS and incubated for further 48 h. 50 μL of MTT reagent (Sig ma-Aldrich, St. Louis, MO) was added to each well and incubated for 1 h at 37°C, after which the absorbance at 490 nm was measured on a microplate reader. The results were expressed as the relative cell viability (%) with respect to control groups treated only with DMEM.

### RNA-Seq Analysis

Total RNA from Ctrl-iPSC-CMs, FC-iPSC-CMs, and corrected FC-iPSC-CMs was extracted using TRIzol reagent (Thermo Fisher Scientific, Waltham, MA) and resuspended in RNase-free water. The preparation of RNA-seq libraries of polyadenylated RNA was conducted using the TruSeq RNA Library Prep Kit v2 (Illumina, San Diego, CA, United States). cDNA libraries were sequenced using an Illumina NextSeq 500 platform. Following the differential expression analysis using DESeq, raw reads were aligned to the human genome using the RNA-Seq Alignment Application on BaseSpace (Illumina). Differentially expressed genes were identified for each sample and used as a query to search for enriched biological processes and pathways using Enrichr tool.^1^ Hierarchical clustering heatmap was generated using Orange Data Mining tool.^2^ Principal component analysis (PCA) was performed on the Log2(fold change) values using Prcomp function in RStudio (1.3.959 version).

### Measurement of GLA Enzyme Activity

10 μl of lysates were added to 50 μl of assay buffer (lysis buffer without Triton X-100) containing 6 mM of *4*-methylumbelliferyl-β-D-galactopyranoside and 117 mM of N-acetyl-D-galactosamine and incubated at 37°C for 1 h. The stop solution (0.4 M glycine, pH 10.8; 70 μl) was added, and the fluorescence was read on a Victor plate reader (PerkinElmer, Waltham, MA) at 355nm excitation and 460nm emission. The enzyme activity was normalized by total amount of protein.

### Measurement of Electrophysiological Activity

All the experimental procedures were conducted as in the previous study (Chou et al., 2017). Briefly, the probe-containing culture dishes (Alpha MED Scientific, Osaka, Japan) were pre-coated 0.1% gelatin overnight and further washed three times by PBS. iPSCs-derived cardiomyocytes were seeded on the pre-coated dishes and the culture medium the day after. The electrical potentials were examined by MED64 multi-electrode array system (Alpha MED Scientific, Osaka, Japan) after the CMs spontaneous beating.

### RT-PCR and qRT-PCR

Total RNA was isolated using the TRIzol reagent (Thermo Fisher Scientific) according to the manufacturer’s instructions. Total RNA was reverse transcribed into cDNA using a SuperScript III Reverse Transcriptase kit (Thermo Fisher Scientific).

The following primer pairs were used: wild type *GLA* (forward TTGGATACTACGACATTGATGCC, reverse GTAT AATTGGGCTTTTGAAAGG), *GLA* IVS4 + 919 G > A mutant (forward GTCCTTGGCCCTGAATAG, reverse GTCCAGCA ACATCAACAATT), *NPPA* (forward TAGAAGATGAGGTC GTGCCC, reverse CGCCCTCAGCTTGCTTTTTA), *ACTC1* (forward TGTGCCAAGATGTGTGACGA, reverse AGGGTCA GGATGCCTCTCTT), *MYL2* (forward GGGCGAGTGAACGT GAAAAA, reverse GTGGTCAGCATTTCCCGAAC), and *MYL7* (forward TCCATGTTTGAACAAGCCCA, reverse AAAGAGC GTGAGGAAGACGG).

## Results

### Preparation and Characterization of PU-PEI_600_-Mal Complexes Loaded With the Plasmids Encoding the Components of CRISPR/Cas9 System

**FIGURE 1:**
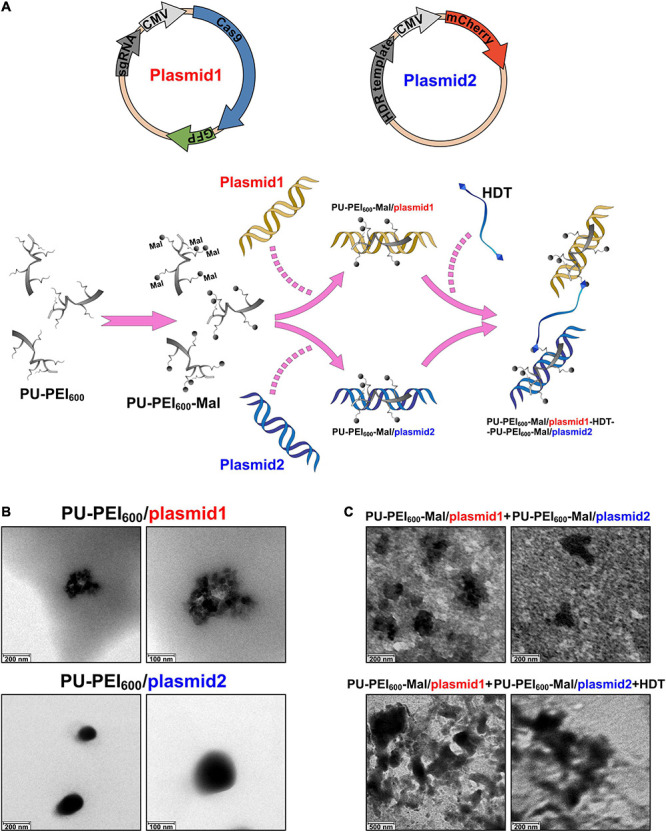
Preparation and characterization of PU-PEI_600_-Mal complexes loaded with the plasmids encoding the components of CRISPR/Cas9 system. **(A)** Schematic outline of experimental design showing the structures of two plasmids (top) and workflow of chemical modifications to generate conjugate complexes for transfection (bottom). **(B)** TEM images of the indicated PU-PEI_600_/DNA complexes. **(C)** TEM images of a mix of unconjugated PU-PEI_600_-Mal/plasmid1 and PU-PEI_600_-Mal/plasmid2 complexes (top) and the same complexes conjugated by HDT (bottom).

Cas9 endonuclease, homology directed repair (HDR) DNA donor template, and sgRNA are the key components of the CRISPR/Cas9 gene editing system. Conventionally, considering the limited cloning capacity of plasmids, the Cas9-encoding gene and DNA donor template are usually loaded in distinct plasmids and transfected separately into target cells. Co-expression of all the components of CRISPR/Cas9 system in a single cell is the major prerequisite for successful editing. In this study, we designed an approach to ensure co-delivery of two plasmids encoding components of CRISPR/Cas9 system utilizing our previously designed polyurethane (PU)-based short branch polyethyleneimine (PEI_600_)-grafted nanoparticles (PU-PEI_600_) (Hung and Cherng, 2015). In such an approach, PU-PEI_600_ was first functionalized by a maleimide (Mal) group to form PU-PEI_600_-Mal (Figure 1A). Two distinct plasmids, one encoding sgRNA, Cas9 and GFP reporter (plasmid1), another encoding DNA template and mCherry reporter (plasmid2) were separately loaded on PU-PEI_600_-Mal particles, and 1,6-hexanedithiol (HDT) was used to covalently conjugate two nanoparticle/DNA formulations (Figure 1A).

PU-PEI_600_ and PU-PEI_600_-Mal were synthesized as described in our previous work and the chemical structures of these polymers were confirmed by FT-IR and ^1^H NMR spectroscopy (Hung and Cherng, 2015). The size of polymer/DNA complexes is affected by a polymer’s capacity to condense DNA. Therefore, to evaluate the efficiency of plasmid1 and plasmid2 DNA condensation by PU-PEI_600_ and PU-PEI_600_-Mal, we measured the sizes of the respective complexes by the dynamic light scattering (DLS). The sizes of PU-PEI_600_/plasmid1 (encoding Cas9) and PU- PEI_600_/plasmid2 (encoding mCherry) prepared at a ratio of 5/1 were around 150 and 90 nm, respectively (Table 1). The grafting of Mal group resulted in larger polymer/DNA complexes, approximately 300 nm of PU-PEI_600_-Mal/plasmid1 and 200 nm of PU-PEI_600_-Mal/plasmid2, which was indicative of less efficient DNA condensation by the modified complexes (Table 1). As was demonstrated by measurement of zeta potentials of PU-PEI_600_ and PU-PEI_600_-Mal, the grafting of Mal resulted in decreased positive charge due to amidation reaction on the 1o amine of PEI_600_ (Table 2). These data indicate that the size of polymer/DNA complexes is affected by structural arrangements on a polymer and by distribution of positive charge density (Hung et al., 2009).

**TABLE 1:** The sizes of different PU-PEI_600_/DNA complex formulations measured by DLS.

**Polymer/DNA complex**	**Size**	**Pdi**
PU-PEI_600_/plasmid1	150 ± 6.21 nm	0.28
PU-PEI_600_/plasmid2	90 ± 10.63 nm	0.20
PU-PEI_600_-Mal/plasmid1	318.9 ± 10.04 nm	0.37
PU-PEI_600_-Mal/plasmid2	228.1 ± 12.44 nm	0.56
PU-PEI_600_-Mal/plasmid1 + PU-PEI_600_-Mal/plasmid2	299.8 ± 22.74 nm	0.55
PU-PEI_600_-Mal/plasmid2 + PU-PEI_600_-Mal/plasmid2 + HDT	546.4 ± 16.46 nm	0.71
		

**TABLE 2:** Zeta potentials of different PU-PEI_600_/DNA complexes.

**Polymer/DNA complex**	**Zeta potential**
PU-PEI_600_/plasmid1	55 mV
PU-PEI_600_/plasmid2	51 mV
PU-PEI_600_-Mal/plasmid1	47.6 ± 0.45 mV
PU-PEI_600_-Mal/plasmid2	42.3 ± 1.05 mV

To conjugate PU-PEI_600_-Mal/plasmid1 and PU-PEI_600_-Mal/plasmid2 complexes for their concomitant delivery into cells, Mal groups were covalently linked via a thioester bond by treatment with HDT. As was shown by DLS, the average size of such conjugated complex was 550 nm, when a molar ratio 1.25/1 of HDT/PU-PEI_600_-Mal was applied (Table 1). Since the average sizes of individual unconjugated complexes were 300 nm and 200 nm, it can be estimated that a conjugate with a size of 550 nm with a broad size distribution (Pdi = 0.7) could consist of two PU-PEI_600_-Mal/DNA complex moieties and the probability to have two distinct complexes in a conjugate is estimated as 50%. Moreover, as was demonstrated by transmission electron microscopy (TEM), the individual PU-PEI_600_/DNA (Figure 1B) and PU-PEI_600_-Mal/DNA complexes not subjected to HDT-mediated crosslinking (Figure 1C) looked like fairly separated particles. In contrast, HDT-treated PU-PEI_600_-Mal/DNA complexes looked significantly more compacted and formed aggregates (conjugates) (Figure 1C).

### Generation and Characterization of Fabry Disease Patient-Specific iPSC-Derived Cardiomyocytes

**FIGURE 2:**
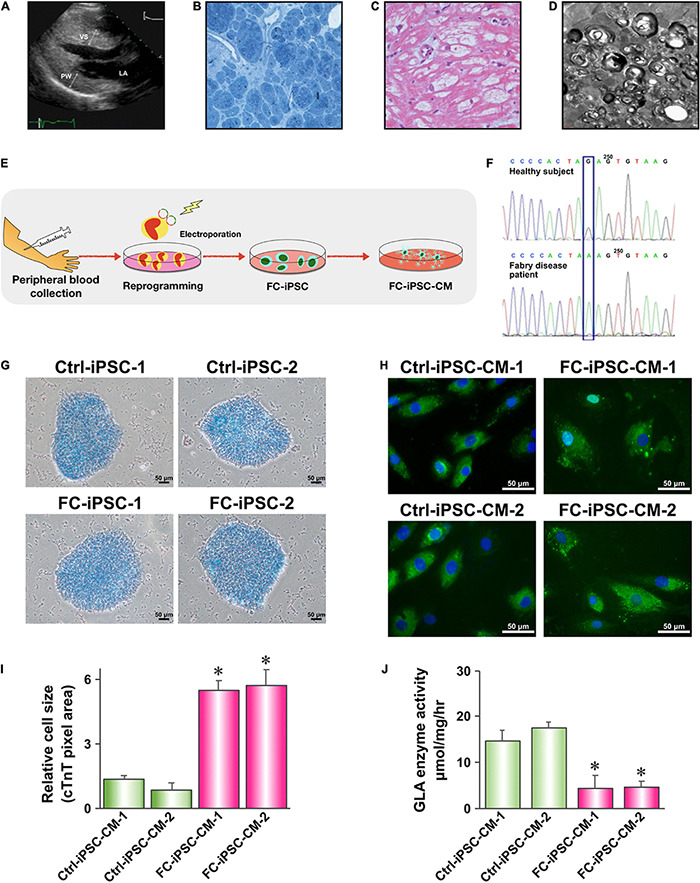
Generation and characterization of Fabry disease patient-specific iPSC-derived cardiomyocytes. **(A)** Transthoracic echocardiogram demonstrating marked ventricular septum (VS) and posterior wall (PW) hypertrophy of the left ventricle. **(B)** H&E staining of a myocardial biopsy sample showing markedly hypertrophic and disorganized myocytes with large perinuclear and sarcoplasmic vacuoles. **(C)** Toluidine blue staining of a myocardial biopsy sample showing accumulation of glycosphingolipids. **(D)** Transmission electron microscopy (TEM) image of a myocardial biopsy sample showing lamellar bodies (zebra bodies) representing lysosomes containing glycolipids. Magnification 60,000×. **(E)** Schematic outline of experimental approach to generate Fabry disease patient-specific iPSC-derived cardiomyocytes. **(F)** Sanger sequencing confirming the presence of IVS4 + 919 G > A mutation in FC-iPSC cell line. **(G)** Alkaline phosphatase staining of Ctrl-iPSC and FC-iPSC clones. **(H)** Immunofluorescent staining of cardiac troponin T (cTnT) in Ctrl-iPSC-CM and FC-iPSC-CM clones at post-induction day 60. **(I)** Quantification of area size of Ctrl-iPSC-CM and FC-iPSC-CM clones. **(J)** Quantification of GLA enzymatic activity in Ctrl-iPSC-CM and FC-iPSC-CM clones. In **(I,J)** mean values from three independent experiments are show with standard deviation error bars, ^∗^*p* < 0.01 (Student’s *t*-test).

In the present study, we sought to use FC-iPSC-CMs as an *in vitro* platform to evaluate the efficacy of CRISPR/Cas9-mediated gene editing on FC-related manifestations, and test the efficiency of PU-PEI_600_-Mal-mediated delivery of CRISPR/Cas9 components. Therefore, iPSCs were obtained by reprogramming T lymphocytes of two patients, who exhibited typical symptoms and clinical signs of FC, including marked ventricular septum and posterior wall hypertrophy of the left ventricle (Figure 2A), markedly hypertrophic and disorganized myocyte structure with large perinuclear and sarcoplasmic vacuoles (Figure 2B), accumulation of glycosphingolipids in the myocardium (Figure 2C), the presence of lamellar bodies (zebra bodies) representing lysosomes containing glycolipids in the myocardium (Figure 2D). FC patient-specific iPSCs (FC-iPSCs) were generated from patients’ T cells by overexpression of Yamanaka factors, and control iPSCs (Ctrl-iPSCs) were generated from two healthy subjects simultaneously (Figure 2E). Both FC-iPSC lines were confirmed to carry the GLA IVS4 + 919 G > A mutation by Sanger sequencing (Figure 2F). All generated FC-iPSC and Ctrl-iPSC clones were positively stained for alkaline phosphatase and exhibited embryonic stem cell-like morphology and other iPSC characteristics as described previously (Chien et al., 2016, 2018; Figure 2G). FC-iPSCs and Ctrl-iPSCs were further subjected to a 40-day cardiomyocyte differentiation protocol (Chien et al., 2016, 2018; Chou et al., 2017) to generate FC-iPSC-CMs and Ctrl-iPSC-CMs, respectively. FC-iPSC-CM and Ctrl-iPSC-CM clones were immunostained positively for cardiac troponin T (cTnT) and image analysis revealed that FC-iPSC-CMs exhibited cellular hypertrophy as compared to Ctrl-iPSC-CMs (Figures 2H,I). Meanwhile, the GLA enzyme activity of FC-iPSC-CMs was significantly lower than that of Ctrl-iPSC-CMs (Figure 2J).

### HDT Conjugation Enhances PU-PEI_600_-Mal-Mediated Co-delivery of CRISPR/Cas9 Components

**FIGURE 3:**
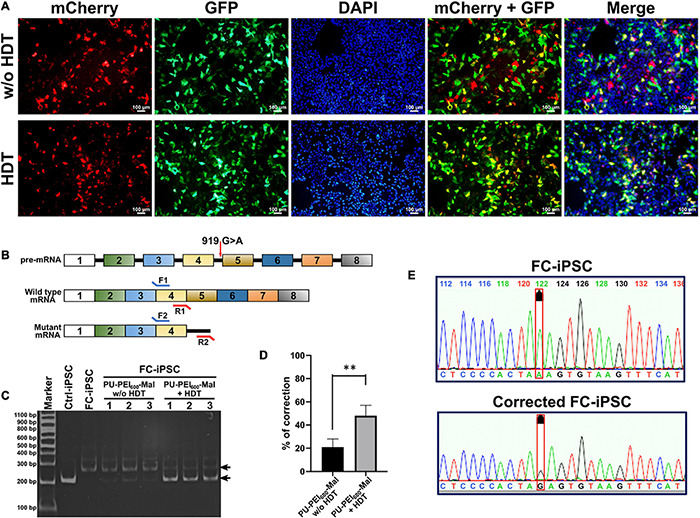
HDT conjugation enhances PU-PEI_600_-Mal-mediated co-delivery of CRISPR/Cas9 components. **(A)** Fluorescence microscopy images of CM-iPSCs treated with the unconjugated mix of PU-PEI_600_-Mal/plasmid1 and PU-PEI_600_-Mal/plasmid2 complexes (top) and HDT-conjugated complexes (bottom) showing the expression of GFP (green) and mCherry (red). **(B)** Schematic outline of *GLA* gene and mRNA products of its wild type and IVS4 + 919 G > A mutant forms with the indicated positions of forward (F) and reverse (R) primers designed to specifically differentiate between them. **(C)** RT-PCR analysis of the expression of wild type (217 bp amplicon) and IVS4 + 919 G > A (272 bp amplicon) *GLA* forms in CM-iPSCs treated with the indicated PU-PEI_600_ CRISPR/Cas9 carrier complexes. **(D)** Quantification of band intensity of RT-PCR electrophoregrams **(C)** and calculated percentage of mutant to wild type correction. Three independent experiments were performed, and mean values are shown with standard deviation error bars. ^∗∗^*p* < 0.001 (Student’s *t*-test). **(E)** Sanger sequencing showing A→G correction of IVS4 + 919 G > A genotype in FC-iPSCs treated with PU-PEI_600_-Mal-HDT carrying CRISPR/Cas9 components (bottom). The sequencing of the original FC-iPSC clone shown on top.

Next, we evaluated the delivery efficiency of unconjugated mix of PU-PEI_600_-Mal/plasmid1 and PU-PEI_600_-Mal/plasmid2 with that of HDT-conjugated complex. As was shown by fluorescence microscopy, HDT-mediated conjugation resulted in markedly more efficient co-expression of GFP and mCherry reporters indicative of co-delivery of plasmid1 and plasmid2 to the same cells (Figure 3A). The simultaneous delivery of plasmid1 and plasmid2 encoding different components of CRISPR/Cas9 system is an absolute requirement for successful editing. Given that IVS4 + 919 G > A mutation results in an alternatively spliced form of GLA mRNA (Ishii et al., 2002), we designed forward and reverse primers to specifically amplify wild type (217 bp amplicon) and mutant (272 bp amplicon) forms (Figure 3B). FC-iPSCs were treated with the polymer/DNA mixture (PU-PEI_600_-Mal/plasmid1 and PU-PEI_600_-Mal/plasmid2 without HDT) and HDT-conjugated polymer/DNA complexes (PU-PEI_600_-Mal/plasmid1-HDT-PU-PEI_600_-Mal/plasmid2) and expression of wild type and mutant forms of GLA mRNA was evaluated by RT-PCR. The results of RT-PCR showed that, comparing with the polymer/DNA mixture without HDT conjugation, HDT enhanced the generation of wild type GLA mRNA with the concomitant decrease of the remnant mutated GLA mRNA (Figures 3C,D). Furthermore, sequence analysis confirmed the A > G gene correction of the IVS4 + 919 G > A mutated genotype in two clones of FC-iPSCs (Figure 3E). Collectively, these data indicated that HDT conjugation could enhance concomitant transfection of two distinct plasmids and, therefore, enhanced the CRISPR/Cas9-mediated correction efficiency.

### PU-PEI_600_-Mal-HDT-Mediated Transfection Is Not Cytotoxic for iPSCs

**FIGURE 4:**
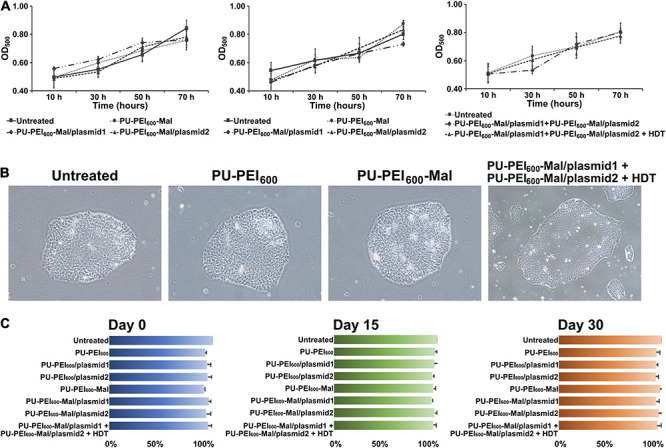
PU-PEI_600_-Mal-HDT-mediated transfection is not cytotoxic for iPSCs. **(A)** MTT cell viability assay showing growth/viability of Ctrl-iPSCs treated with the indicated formulations of PU-PEI_600_/DNA complexes. Data shown as means from three independent experiments with standard deviation error bars. **(B)** Bright-field images of Ctrl-iPSCs treated with the indicated PU-PEI_600_/DNA complexes. **(C)** Comparison of cell viability between untreated Ctrl-iPSCs and Ctrl-iPSCs treated with the indicated PU-PEI_600_/DNA complexes at different time points of cardiac differentiation (days 0, 15, and 30). Data expressed as mean percentages relative to “Untreated” from three independent experiments with standard deviation error bars.

Cytotoxicity is a critical issue that hinders the success of gene delivery. Previously, our data revealed that PU-PEI_600_/DNA complexes were not cytotoxic for COS-7 cells till a ratio of 100/1 (w/w). In addition, HDT-conjugated PU-PEI_600_-Mal/DNA complexes only showed a mild effect on the viability of COS-7 cells when HDT/PU-PEI_600_-Mal/DNA molar ratios were below 5/1 (w/w) (Hung and Cherng, 2015). To evaluate whether PU-PEI_600_, PU-PEI_600_-Mal, or HDT-conjugated PU-PEI_600_-Mal affect the viability of Ctrl-iPSCs, we measured the growth of Ctrl-iPSCs and the viability of Ctrl-iPSC-CMs after transfection with the indicated polymer/DNA complexes. As was shown by MTT assay, there was no significant difference in cell growth of parental Ctrl-iPSCs treated with PU-PEI_600_ free of DNA or loaded with plasmid 1 or plasmid2 (Figure 4A). A parallel experiment also showed that the HDT linker did not modify the growth of Ctrl-iPSCs after all indicated transfections (Figure 4A, right). Meanwhile, all of polymers tested in this study did not affect the ability of iPSCs to form colonies (Figure 4B). In addition, the transfection with any polymer/DNA complex did not affect the ability of Ctrl-iPSCs to undergo embryoid body formation and cardiac differentiation (data not shown). Furthermore, the cell viability was not changed by any given treatment at post-induction days 0, 15, or 30 (Figure 4C). Taken together, these data revealed that neither PU-PEI_600_ *per se*, Mal-functionalized PU-PEI_600_, nor HDT-conjugated PU-PEI_600_ nanoparticles affected the regular growth of Ctrl-iPSCs and their ability for cardiac differentiation.

### Reversal of Cardiomyocyte Hypertrophy by PU-PEI_600_-Mal-HDT-Delivered CRISPR/Cas9-Mediated Genetic Correction in FC-iPSC-CMs

**FIGURE 5:**
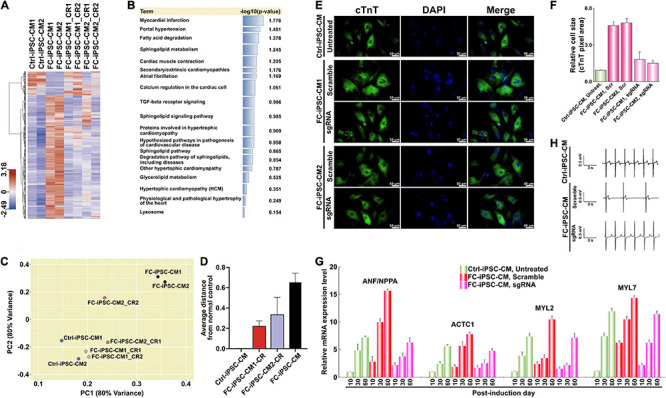
Reversal of cardiomyocyte hypertrophy by PU-PEI_600_-Mal-HDT-delivered CRISPR/Cas9-mediated genetic correction in FC-iPSC-CMs. **(A)** Hierarchical clustering heatmap showing gene expression profiles in wild type Ctrl-iPSC-CMs, uncorrected FC-iPSC-CMs and corrected FC-iPSC-CMs (FC-iPSC-CM_CRs). For each group, the clones derived from two different individuals were analyzed (CM1 and CM2). For each patient-specific clone (CM1 and CM2), two individual corrected clones (CR1 and CR2) were analyzed. **(B)** Gene set enrichment analysis (Enrichr) performed on the dysregulated genes in FC-iPSC-CMs that were repaired by the CRISPR/Cas9-mediated genetic correction. **(C)** Principal component analysis showing that the global gene expression pattern of FC-iPSC-CMs with CRISPR/Cas9-mediated genetic correction is closer to the gene signature of Ctrl-iPSC-CMs rather than that of uncorrected FC-iPSC-CMs. **(D)** The average distance of FC-iPSC-CMs and FC-iPSC-CMs with CRISPR/Cas9-mediated genetic correction from normal control. **(E)** Immunofluorescent staining of cTnT in Ctrl-iPSC-CMs and 2 clones of FC-iPSC-CMs derived from FC-iPSCs treated with scramble sgRNA (negative control) and subjected to GLA editing (sgRNA). Nuclei stained with DAPI. **(F)** Quantification of area size of Ctrl-iPSC-CM and FC-iPSC-CM clones. The results are mean values from three independent experiments with standard deviation error bars. **(G)** Electrical conductivity measurement of the indicated groups of CMs using MED64 microelectrode system. **(H)** qRT-PCR analysis of the expression of selected cardiac hypertrophy-associated genes at different time points of differentiation of unedited (untreated and scramble) and edited (sgRNA) FC-iPSCs to FC-iPSC-CMs.

Along with the observations that HDT-conjugated Mal-functionalized PU-PEI_600_ promoted dual DNA transfection and enhanced CRISPR/Cas9-mediated gene correction, we sought to further examine whether this enhanced gene correction could rescue FC-associated manifestations. First, we subjected Ctrl-iPSC-CMs, FC-iPSC-CMs, and FC-iPSC-CMs with CRISPR/Cas9-mediated gene correction (post-induction day 20) to RNA-Seq analysis. Indeed, the gene expression profiles of corrected (CR) FC-iPSC-CM clones were similar to those of Ctrl-iPSC-CMs, but rather different from uncorrected FC-iPSC-CMs (Figure 5A). Furthermore, gene set enrichment analysis revealed that dysregulated genes in FC-iPSC-CMs that were rescued by the CRISPR/Cas9-mediated genetic correction, were enriched in the pathways related to myocardial infarction, portal hypertension, fatty acid degradation, sphingolipid metabolism, cardiac muscle contraction, secondary/extrinsic cardiomyopathies, atrial fibrillation, calcium regulation in the cardiac cell, TGF-beta receptor signaling, sphingolipid signaling pathway, proteins involved in hypertrophic cardiomyopathy, hypothesized pathways in pathogenesis of cardiovascular disease, sphingolipid pathway, degradation pathway of sphingolipids, other hypertrophic cardiomyopathy, glycerolipid metabolism, hypertrophic cardiomyopathy, physiological and pathological hypertrophy of the heart, and lysosome (Figure 5B). Principal component analysis further revealed that the global gene expression pattern of FC-iPSC-CMs with CRISPR/Cas9-mediated genetic correction was closer to that of Ctrl-iPSC-CMs than FC-iPSC-CMs (Figures 5C,D).

Cardiomyocyte hypertrophy is one of the major clinical manifestations in FC patients with IVS4 + 919 G > A mutation. To examine whether CRISPR/Cas9-mediated genetic correction of IVS4 + 919 G > A could prevent the development of cardiomyocyte hypertrophy in FC-iPSC-CMs that carried IVS4 + 919 G > A, the transfected FC-iPSCs and untreated Ctrl-iPSCs were directed to cardiac differentiation and the cardiomyocyte size was measured by quantification of the cTnT-positive area in FC-iPSC-CMs. Compared with that of Ctrl-iPSC-CMs, FC-iPSC-CMs that carried IVS4 + 919 G > A consistently exhibited the enlarged cell size at post-induction days 30–60, whereas FC-iPSC-CMs subjected to CRISPR/Cas9-mediated genetic correction did not undergo cell hypertrophy (Figures 5E,F). Several genes, including NPPA (natriuretic peptide A), ACTC1 (alpha-cardiac actin), MYL2 (myosin regulatory light chain 2), and MYL7 (myosin regulatory light chain 7), have been previously implicated in cardiac hypertrophy (Lan et al., 2013). qRT-PCR analysis confirmed the upregulation of these cardiac hypertrophy-associated genes in the time course of cardiac induction (Figure 5G). All of these hypertrophy-associated genes were upregulated in all groups of CMs, however, they were much less efficiently upregulated in CRISPR/Cas9-corrected FC-iPSC-CMs as compared to Ctrl-iPSC-CMs and FC-iPSC-CMs treated with scramble control formulation (Figure 5G). One of the important features of Fabry disease is the electric conduction abnormalities. Therefore, we applied MED64 array system to measure the electrical conductivity of the CMs. Comparing with Ctrl-iPSC-derived CMs, Fabry iPSC-derived CMs exhibited impaired conductivity. No significant difference was observed between Ctrl-iPSC-derived CMs and corrected iPSC-derived CMs (Figure 5H). These results indicate that CRISPR/Cas9-mediated genetic correction of IVS4 + 919 G > A effectively prevented the cardiomyocyte hypertrophy in FC-iPSC-CMs and prevented the impairment of conductivity.

### PU-PEI_600_-Mal-HDT-Mediated CRISPR/Cas9 Gene Correction Abolishes the Gb3 Deposition in FC-iPSC-CMs

**FIGURE 6:**
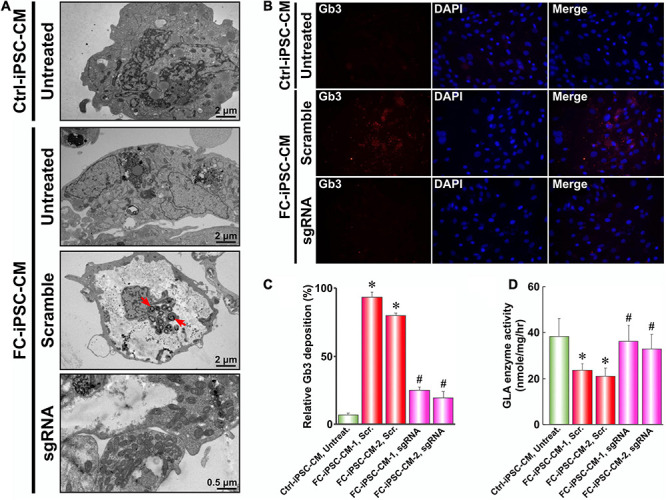
PU-PEI_600_-Mal-HDT-mediated CRISPR/Cas9 gene correction abolishes the Gb3 deposition in FC-iPSC-CMs. **(A)** TEM images of Ctrl-iPSC-CMs and FC-iPSC-CMs with (sgRNA) or without (scramble and untreated) *GLA* gene editing. Red arrows indicate lysosomal Gb3 depositions. **(B)** Immunostaining with the antibody against Gb3 of Ctrl-iPSC-CMs and FC-iPSC-CMs with (sgRNA) or without (scramble) *GLA* gene editing. Nuclei stained with DAPI. **(C)** Quantification of immunofluorescent signal of Gb3 in Ctrl-iPSC-CMs and two cell lines of FC-iPSC-CMs without (Scr.) and with (sgRNA) *GLA* gene editing. Data expressed as mean values relative to Ctrl-iPSC-CM from three independent experiments with standard deviation error bars. ^∗^*p* < 0.01 vs. Ctrl-iPSC-CM Untreated, #*p* < 0.01 vs. FC-iPSC-CM-1 Scr (Student’s *t*-test) **(D)** GLA enzyme activity in Ctrl-iPSC-CMs and two cell lines of FC-iPSC-CMs without (Scr.) and with (sgRNA) *GLA* gene editing. Mean values from three independent experiments are shown with standard deviation error bars. ^∗^*p* < 0.01 vs. Ctrl-iPSC-CM Untreated, #*p* < 0.01 vs. FC-iPSC-CM-1 Scr (Student’s *t*-test).

Since intramyocardial Gb3 accumulation resulting from GLA deficiency is the landmark of the FC and the milestone for monitoring the efficacy of enzyme replacement therapy, we therefore evaluated the lysosomal abnormalities and the magnitude of Gb3 accumulation in FC-iPSC-CMs by TEM. After the dual transfection-mediated gene correction and the induction of cardiac differentiation, transfected FC-iPSC-CMs were subjected to TEM inspection. Comparing with untreated Ctrl-iPSC-CMs, untreated FC-iPSC-CMs and FC-iPSC-CMs transfected with the scramble sequence exhibited widespread hypertrophic and vacuolated features with electron-dense concentric lamellar bodies in the cytosol at day 40 post-induction (Figure 6A). Remarkably, FC-iPSC-CMs subjected to PU-PEI_600_-Mal-HDT-mediated CRISPR/Cas9 gene correction did not reveal the formation of Gb3-enriched lamellar bodies (Figure 6A). We further used immunofluorescence staining to validate this observation and found a strong intensity of Gb3 signal within FC-iPSC-CMs transfected with a scramble sequence (Figures 6B,C). Consistently with the findings of TEM, Gb3 accumulation was not observed in FC-iPSC-CMs subjected to CRISPR/Cas9-mediated gene correction (Figures 6B,C). Since GLA is the enzyme responsible for the cleavage of Gb3, we also evaluated the GLA activity in Ctrl-iPSC-CMs, FC-iPSC-CMs transfected with scramble sequence, and FC-iPSC-CMs subjected to CRISPR/Cas9-mediated gene correction. Comparing with that of Ctrl-iPSC-CMs, the basal GLA activity was reduced in FC-iPSC-CMs transfected with a scramble sequence, while CRISPR/Cas9-mediated gene correction restored this activity (Figure 6D). Collectively, these data indicated that CRISPR/Cas9-mediated genetic correction of IVS4 + 919G > A mutation could prevent the development of Gb3 depositions through restoration of GLA enzyme activity in FC-iPSC-CMs.

## Discussion

In Taiwan, Fabry cohorts with IVS4 + 919 G > A mutation usually exhibit late-onset cardiomyopathy. Enzyme replacement therapy (ERT) with routine administration of recombinant human alpha-galactosidase A (rhα-GLA) is currently the only available and effective treatment for the clearance of Gb3 deposition in patients. Previously, we have reported that ERT could reduce the severity of FC and suppress the concomitant elevation of proinflammatory cytokines (Chen et al., 2016). However, in some cases, the treatment outcome of FC was dismal, especially in patients who had developed myocardial fibrosis (Yousef et al., 2013). In addition, the short half-life of rhα-GLA and the demand for life-long continuous administration also hinder the bioavailability of rhα-GLA treatment in FC patients. Considerable efforts have been made for the past decades and shown the promising potential of gene therapy in the treatment of inherited diseases, such as adenosine deaminase deficiency, familial hypercholesterolemia, and cystic fibrosis (Knoell and Yiu, 1998). We have recently used nanotechnology-based gene therapeutic strategies to treat experimental models of X-linked juvenile retinoschisis (Chou et al., 2020), to enhance the efficacy of chemotherapy in cultured hepatoma cells (Tsai et al., 2019), or to enhance the hepatic differentiation (Chen et al., 2013). Endogenous alpha-galactosidase A, encoded by the GLA gene, is known as the key enzyme responsible for Gb3 cleavage. Direct knockdown of GLA was shown to mimic the pathological features of FC in HEK-293T cells (Song et al., 2016) and in embryonic stem cells (Song et al., 2019), corroborating the pivotal role of GLA in FC pathogenesis. The common GLA IVS4 + 919 G > A mutation was extensively accepted to be the cause of the aberrant alternative splicing that contributes to the phenotypes in a majority of Taiwan FC cohorts (Ishii et al., 2002). Therefore, in addition to the currently available ERT, correction of the mutated genes may represent an ideal non-viral solution for the treatment of FC with IVS4 + 919 G > A mutation.

CRISPR/Cas9-mediated gene editing has shown great promises as a tool for gene manipulations, including gene mutation, deletion, and correction in human cells. However, the limited cloning capacity of plasmids is the hurdle for multiple gene delivery. Cas9 gene and HDR DNA template, the two major components of the CRISPR/Cas9 gene editing system, are usually inserted into distinct plasmids and separately transfected into the target cells. The resultant low probability of the concurrent expression of Cas9 and DNA template may reduce the efficiency of CRISPR/Cas9-mediated gene correction and its application in gene therapy. Interestingly, the thiol-maleimide conjugation has been widely applied in the field of bioconjugation (Aktan et al., 2017). To improve the low efficiency of co-transfection, we used a Mal functionalization and HDT-conjugation-based strategy to modify the PU-PEI_600_-mediated gene delivery system. By means of this method, such HDT-conjugated Mal-functionalized polymers enhanced the concurrent delivery of Cas9, DNA template, and sgRNA, leading to the effective A→G gene correction of GLA mutation in FC-iPSCs (Figure 3). After the gene editing and the employment of cardiac induction protocols, the cardiomyocyte hypertrophy, lysosomal Gb3 deposition, electrical conductivity defect and defective GLA enzymatic activity were all repaired in the corrected clones of FC-iPSC-CMs (Figures 5, 6). Such HDT-conjugated Mal-functionalized non-viral delivery system was characterized by high transfection efficiency and low cytotoxicity in FC-iPSCs and effectively drove the CRISPR/Cas9-based gene correction with high efficiency (Figures 3, 4). Collectively, this non-viral polymer with maleimide-HDT conjugates may serve as an ideal dual delivery system that could facilitate effective and precise CRISPR/Cas9-based genomic correction.

Recent advances in iPSC technologies and the generation of patient-derived iPSCs have generated novel opportunities for regenerative medicine, personalized therapy, and the modeling of hereditary diseases. We have used patient-specific iPSC-derived lineages to model hereditary diseases, including X-linked juvenile retinoschisis (Huang et al., 2019), Leber’s hereditary optic neuropathy (Yang et al., 2020b), Best dystrophy (Lin et al., 2019), and Fabry cardiomyopathy (Chien et al., 2016, 2018). Similar with the features of iPSC-CMs derived from familial hypertension patients (Lan et al., 2013), our studies revealed that FC-iPSC-CMs also chronologically exhibited the typical phenotypes of FC (Chien et al., 2016, 2018). Since differentiated cells are unable to proliferate or differentiate, the efficacy of gene correction to directly repair the disease phenotypes in terminally differentiated cells with an inherited mutation remains questionable. Thus, patient-derived undifferentiated iPSCs or progenitors may be a better choice to be targeted for gene correction. The efficacy of genetic correction can be examined after implantation into *in vivo* experimental model(s) or transplantation into the patients. For example, Maxwell et al. subjected diabetic patient-specific stem cell-derived β cells to CRISPR/Cas9 gene correction, and these corrected cells were able to reverse the pre-existing streptozocin-induced diabetes after transplantation into mice (Maxwell et al., 2020). Ou et al. used CRISPR/Cas9 to correct β-thalassemia mutations in patient-specific iPSCs and then differentiated these corrected iPSCs into hematopoietic stem cells (HSCs). These corrected iPSC-derived HSCs did not carry tumorigenic potential after implantation into immunodeficient mice (Ou et al., 2016). Recent reports further demonstrated that HSCs with genetic editing could offer therapeutic potential for diseases of the blood and immune system and the HSC-based genome-editing field is ready for entering the clinical trials (Dever and Porteus, 2017). For iPSC-CMs, it was reported that transplanted human iPSC-CMs could improve myocardial functions and alleviate ventricular remodeling in rat hearts (Guan et al., 2020). Another study used iPSC-CMs and mesenchymal stem cells to generate micro-tissues for cardiac cell replacement therapy *in vivo* (Sahito et al., 2019). These findings raised the possibilities that gene-edited patient-specific iPSC-CMs can be used as cell resources for intramyocardial transplantation to repair the structural or functional defect in FC patients.

## Data Availability Statement

The raw data supporting the conclusions of this article will be made available by the authors, without undue reservation.

## Ethics Statement

The studies involving human participants were reviewed and approved by Institutional Review Board (IRB) of Taipei Veterans General Hospital (TVGH). The patients/participants provided their written informed consent to participate in this study.

## Author Contributions

C-SC, Y-YL, S-JC, T-XW, P-HT, and HL: investigation. YC, AY, and H-BL: the manuscript writing. Y-PY, M-LW, Y-CJ, and T-IH: project planning and direct supervision. J-YC and C-YW: project supervision and funding acquisition. All authors contributed to the article and approved the submitted manuscript.

## Conflict of Interest

The authors declare that the research was conducted in the absence of any commercial or financial relationships that could be construed as a potential conflict of interest.

## Publisher’s Note

All claims expressed in this article are solely those of the authors and do not necessarily represent those of their affiliated organizations, or those of the publisher, the editors and the reviewers. Any product that may be evaluated in this article, or claim that may be made by its manufacturer, is not guaranteed or endorsed by the publisher.

## Footnotes

1
https://maayanlab.cloud/Enrichr/

2
https://orangedatamining.com/

## References

[B1] Aktan B. Chambre L. Sanyal R. Sanyal A. (2017). Clickable” nanogels via thermally driven self-assembly of polymers: facile access to targeted imaging platforms using thiol-maleimide conjugation. *Biomacromolecules* 18 490–497. 10.1021/acs.biomac.6b01576 28052673

[B2] Boulaiz H. Marchal J. A. Prados J. Melguizo C. Aranega A. (2005). Non-viral and viral vectors for gene therapy. *Cell. Mol. Biol.* 51 3–22. 16171561

[B3] Chen K. H. Chien Y. Wang K. L. Leu H. B. Hsiao C. Y. Lai Y. H. (2016). Evaluation of proinflammatory prognostic biomarkers for Fabry cardiomyopathy with enzyme replacement therapy. *Can. J. Cardiol.* 32 1221.e1–1221.e9. 10.1016/j.cjca.2015.10.033 26919792

[B4] Chen W. Tsai P. H. Hung Y. Chiou S. H. Mou C. Y. (2013). Nonviral cell labeling and differentiation agent for induced pluripotent stem cells based on mesoporous silica nanoparticles. *ACS Nano* 7 8423–8440. 10.1021/nn401418n 24063246

[B5] Chien Y. Chien C. S. Chiang H. C. Huang W. L. Chou S. J. Chang W. C. (2016). Interleukin-18 deteriorates Fabry cardiomyopathy and contributes to the development of left ventricular hypertrophy in Fabry patients with GLA IVS4 + 919 G > A mutation. *Oncotarget* 7 87161–87179. 10.18632/oncotarget.13552 27888626 PMC5349979

[B6] Chien Y. Chou S. J. Chang Y. L. Leu H. B. Yang Y. P. Tsai P. H. (2018). Inhibition of arachidonate 12/15-lipoxygenase improves alpha-galactosidase efficacy in iPSC-Derived Cardiomyocytes from Fabry patients. *Int. J. Mol. Sci.* 19:1480. 10.3390/ijms19051480 29772700 PMC5983630

[B7] Chou S. J. Yang P. Ban Q. Yang Y. P. Wang M. L. Chien C. S. (2020). Dual supramolecular nanoparticle vectors enable CRISPR/Cas9-mediated knockin of retinoschisin 1 Gene-A potential non-viral therapeutic solution for X-linked juvenile retinoschisis. *Adv. Sci.* 7:1903432. 10.1002/advs.201903432 32440478 PMC7237855

[B8] Chou S. J. Yu W. C. Chang Y. L. Chen W. Y. Chang W. C. Chien Y. (2017). Energy utilization of induced pluripotent stem cell-derived cardiomyocyte in Fabry disease. *Int. J. Cardiol.* 232 255–263. 10.1016/j.ijcard.2017.01.009 28082092

[B9] Dever D. P. Porteus M. H. (2017). The changing landscape of gene editing in hematopoietic stem cells: a step towards Cas9 clinical translation. *Curr. Opin. Hematol.* 24 481–488. 10.1097/moh.0000000000000385 28806273 PMC5766279

[B10] Ding Q. Strong A. Patel K. M. Ng S. L. Gosis B. S. Regan S. N. (2014). Permanent alteration of PCSK9 with *in vivo* CRISPR-Cas9 genome editing. *Circ. Res.* 115 488–492. 10.1161/circresaha.115.304351 24916110 PMC4134749

[B11] Eng C. M. Niehaus D. J. Enriquez A. L. Burgert T. S. Ludman M. D. Desnick R. J. (1994). Fabry disease: twenty-three mutations including sense and antisense CpG alterations and identification of a deletional hot-spot in the alpha-galactosidase A gene. *Hum. Mol. Genet.* 3 1795–1799. 10.1093/hmg/3.10.1795 7531540

[B12] Guan X. Xu W. Zhang H. Wang Q. Yu J. Zhang R. (2020). Transplantation of human induced pluripotent stem cell-derived cardiomyocytes improves myocardial function and reverses ventricular remodeling in infarcted rat hearts. *Stem Cell Res. Ther.* 11:73. 10.1186/s13287-020-01602-0 PMC7033912 32085809

[B13] Hsu T. R. Sung S. H. Chang F. P. Yang C. F. Liu H. C. Lin H. Y. (2014). Endomyocardial biopsies in patients with left ventricular hypertrophy and a common Chinese later-onset fabry mutation (IVS4 + 919G > A). *Orphanet J. Rare Dis.* 9:96. 10.1186/1750-1172-9-96 24980630 PMC4100491

[B14] Huang K. C. Wang M. L. Chen S. J. Kuo J. C. Wang W. J. Nhi Nguyen P. N. (2019). Morphological and molecular defects in human three-dimensional retinal organoid model of X-linked juvenile retinoschisis. *Stem Cell Reports* 13 906–923. 10.1016/j.stemcr.2019.09.010 31668851 PMC6895767

[B15] Hung W. C. Cherng J. Y. (2015). Maleimide-Functionalized PEI600 Grafted Polyurethane: Synthesis, Nano-Complex Formation with DNA and Thiol-Conjugation of the Complexes for Dual DNA Transfection. *Polymers* 7 2131–2145. 10.3390/polym7101503

[B16] Hung W. C. Shau M. D. Kao H. C. Shih M. F. Cherng J. Y. (2009). The synthesis of cationic polyurethanes to study the effect of amines and structures on their DNA transfection potential. *J. Control. Release* 133 68–76. 10.1016/j.jconrel.2008.09.082 18930774

[B17] Ishii S. Nakao S. Minamikawa-Tachino R. Desnick R. J. Fan J. Q. (2002). Alternative splicing in the alpha-galactosidase A gene: increased exon inclusion results in the Fabry cardiac phenotype. *Am. J. Hum. Genet.* 70 994–1002. 10.1086/339431 11828341 PMC379133

[B18] Karras A. De Lentdecker P. Delahousse M. Debauchez M. Tricot L. Pastural M. (2008). Combined heart and kidney transplantation in a patient with Fabry disease in the enzyme replacement therapy era. *Am. J. Transplant.* 8 1345–1348. 10.1111/j.1600-6143.2008.02245.x 18522550

[B19] Knoell D. L. Yiu I. M. (1998). Human gene therapy for hereditary diseases: a review of trials. *Am. J. Health Syst. Pharm.* 55 899–904. 10.1093/ajhp/55.9.899 9588249

[B20] Lan F. Lee A. S. Liang P. Sanchez-Freire V. Nguyen P. K. Wang L. (2013). Abnormal calcium handling properties underlie familial hypertrophic cardiomyopathy pathology in patient-specific induced pluripotent stem cells. *Cell Stem Cell* 12 101–113. 10.1016/j.stem.2012.10.010 23290139 PMC3638033

[B21] Lee S. H. Li C. F. Lin H. Y. Lin C. H. Liu H. C. Tsai S. F. (2014). High-throughput detection of common sequence variations of Fabry disease in Taiwan using DNA mass spectrometry. *Mol. Genet. Metab.* 111 507–512. 10.1016/j.ymgme.2014.02.004 24613481

[B22] Lian X. Zhang J. Azarin S. M. Zhu K. Hazeltine L. B. Bao X. (2013). Directed cardiomyocyte differentiation from human pluripotent stem cells by modulating Wnt/beta-catenin signaling under fully defined conditions. *Nat. Protoc.* 8 162–175. 10.1038/nprot.2012.150 23257984 PMC3612968

[B23] Lin T. C. Lin Y. Y. Hsu C. C. Yang Y. P. Yang C. H. Hwang D. K. (2019). Nanomedicine-based Curcumin Approach Improved ROS Damage in Best Dystrophy-specific Induced Pluripotent Stem Cells. *Cell Transplant.* 28 1345–1357. 10.1177/0963689719860130 31313605 PMC6802151

[B24] Maxwell K. G. Augsornworawat P. Velazco-Cruz L. Kim M. H. Asada R. Hogrebe N. J. (2020). Gene-edited human stem cell-derived beta cells from a patient with monogenic diabetes reverse preexisting diabetes in mice. *Sci. Transl. Med.* 12:eaax9106. 10.1126/scitranslmed.aax9106 32321868 PMC7233417

[B25] Ou Z. Niu X. He W. Chen Y. Song B. Xian Y. (2016). The combination of CRISPR/Cas9 and iPSC technologies in the gene therapy of human beta-thalassemia in mice. *Sci. Rep.* 6:32463. 10.1038/srep32463 PMC5007518 27581487

[B26] Sahito R. G. A. Sheng X. Maass M. Mikhael N. Hamad S. Heras-Bautista C. O. (2019). *In Vitro* grown micro-tissues for cardiac cell replacement therapy *in Vivo*. Cell. Physiol. Biochem. 52 1309–1324. 10.33594/000000092 31050280

[B27] Song H. Y. Chiang H. C. Tseng W. L. Wu P. Chien C. S. Leu H. B. (2016). Using CRISPR/Cas9-mediated GLA gene knockout as an in vitro drug screening model for Fabry disease. *Int. J. Mol. Sci.* 17:2089. 10.3390/ijms17122089 27983599 PMC5187889

[B28] Song H. Y. Chien C. S. Yarmishyn A. A. Chou S. J. Yang Y. P. Wang M. L. (2019). Generation of GLA-knockout human embryonic stem cell lines to model autophagic dysfunction and exosome secretion in Fabry disease-associated hypertrophic cardiomyopathy. *Cells* 8:327. 10.3390/cells8040327 30965672 PMC6523555

[B29] Tsai P. H. Wang M. L. Chang J. H. Yarmishyn A. A. Nhi Nguyen P. N. Chen W. (2019). Dual Delivery of HNF4alpha and Cisplatin by Mesoporous Silica Nanoparticles Inhibits Cancer Pluripotency and Tumorigenicity in Hepatoma-Derived CD133-Expressing Stem Cells. *ACS Appl. Mater. Interfaces* 11 19808–19818. 10.1021/acsami.9b04474 31066542

[B30] Weidemann F. Niemann M. Breunig F. Herrmann S. Beer M. Stork S. (2009). Long-term effects of enzyme replacement therapy on fabry cardiomyopathy: evidence for a better outcome with early treatment. *Circulation* 119 524–529. 10.1161/circulationaha.108.794529 19153271

[B31] Yang T. C. Chang C. Y. Yarmishyn A. A. Mao Y. S. Yang Y. P. Wang M. L. (2020a). Carboxylated nanodiamond-mediated CRISPR-Cas9 delivery of human retinoschisis mutation into human iPSCs and mouse retina. *Acta Biomater.* 101 484–494. 10.1016/j.actbio.2019.10.037 31672582

[B32] Yang T. C. Yarmishyn A. A. Yang Y. P. Lu P. C. Chou S. J. Wang M. L. (2020b). Mitochondrial transport mediates survival of retinal ganglion cells in affected LHON patients. *Hum. Mol. Genet.* 29 1454–1464. 10.1093/hmg/ddaa063 32277753

[B33] Yousef Z. Elliott P. M. Cecchi F. Escoubet B. Linhart A. Monserrat L. (2013). Left ventricular hypertrophy in Fabry disease: a practical approach to diagnosis. *Eur. Heart J.* 34 802–808. 10.1093/eurheartj/ehs16 22736678 PMC3596758

